# Inhibitory Effects and Mechanism of the Natural Compound Diaporthein B Extracted from Marine-Derived Fungi on Colon Cancer Cells

**DOI:** 10.3390/molecules27092944

**Published:** 2022-05-05

**Authors:** Peihua Tang, Dandan Liu, Zheli Wu, Hui Cui, Ren Zhang, Zaoyuan Kuang

**Affiliations:** 1 School of Basic Medical Sciences, Guangzhou University of Chinese Medicine, Guangzhou 510006, China; 021435@gzucm.edu.cn (P.T.); 20191101023@stu.gzucm.edu.cn (D.L.); wzl2006@gzucm.edu.cn (Z.W.); 2 Research Center of Integrative Medicine, School of Basic Medical Sciences, Guangzhou University of Chinese Medicine, Guangzhou 510006, China; 3 School of Pharmaceutical Sciences, Guangzhou University of Chinese Medicine, Guangzhou 510006, China; cuihui@gzucm.edu.cn

**Keywords:** colon cancer, DTB, mitochondrial apoptosis pathway, Hippo, YAP

## Abstract

This study aimed to investigate the inhibitory effects and mechanism of diaporthein B (DTB), a natural compound extracted from the fungus Penicillium sclerotiorum GZU-XW03-2, on human colon cancer cells. The inhibitory effect of DTB at different concentrations on the proliferation of colon cancer cells HCT116 and LOVO was detected at 24 and 48 h. The effect of cell migration and clone formation ability were detected by cell scratch and plate cloning experiments. Morphological changes were observed by Hoechst 33342 and Annexin-V/PI staining, and flow cytometry was used to detect the proportion of apoptotic cells. DTB significantly inhibited colon cancer cell proliferation, migration, and apoptosis in a dose-dependent manner without significant effects on normal colonic epithelial cells NCM460. The IC50 inhibition effect can be achieved after treatment with 3 μmol/L DTB for 24 h. Compared with the blank group, the migration and clonal-forming ability of colon cancer cells in the DTB group was significantly decreased (*p* < 0.01), while the apoptotic cells were significantly increased (*p* < 0.01) in a concentration-dependent manner. DTB can inhibit the proliferation and migration of human colon cancer cells HCT116 and LOVO and promote the apoptosis of human colon cancer cells.

## 1. Introduction

**Figure 1:**
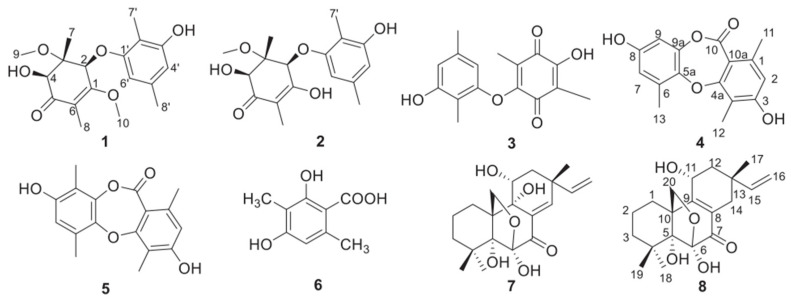
Chemical structures of **1**–**8**; the chemical structure of DTB: C20H28O6, mass: 364.19.

Cancer has always been a difficult problem that mankind is yet to solve. According to statistics, the number of new cancer cases worldwide in 2020 was approximately 19.29 million, of which 4.57 million were in China. According to the 2019 National Cancer Quality Control Center statistics, colon cancer, one of the most common malignant tumors, continuously increases in incidence, ranking third. Colon cancer accounts for approximately 1 in 10 cases and deaths [1]. Surgery, radiotherapy, and chemotherapy are the main clinical treatment methods for colon cancer. However, the five-year survival rate of patients with middle and advanced colon cancer is still low and the postoperative recurrence rate is high; the same is true for the mortality rate [2]. In tumor treatment, due to multi-channel, multi-target, and multi-link characteristics, traditional Chinese medicine is an important part of current anti-tumor treatment [3]. Traditional Chinese medicine preparations and extracts can act on tumor cells through different channels and multiple targets to inhibit the proliferation and migration of tumor cells, improve the self immunity of patients, and promote tumor cell apoptosis. The combination of radiotherapy and chemotherapy can improve the curative effect and reduce the adverse reactions caused by radiotherapy and chemotherapy [4]. In the treatment of colon cancer, drugs for reducing heat and dampness, removing blood stasis, detoxification, dispersing knots, and eliminating symptoms are often used. Clinically, fungus medicine is widely used, such as Poria, Cordyceps, etc. As one of the important members of marine microorganisms, marine fungi have great potential for exploration. The special ecological environment of the ocean has created a special metabolic mechanism of marine microorganisms, which in turn produces metabolites with novel structures and unique activities, which are important sources of new marine natural products. Discovering drug lead compounds from marine biological resources has become an important development trend in new drug research. These new compounds have significant biological and pharmacological properties, such as anti-tumor, hypolipidemic, anti-inflammatory, etc. [5,6,7]. Three new compounds **1**, **4** and **8**, as well as five known compounds **2**, **3**, **5**, **6**, **7**, were isolated and identified from the EtOAc extract of the fermentation broth of the marine fungus Penicillium sclerotiorum GZU-XW03-2 was isolated from the intestinal tract of the Onchidium struma and collected from Cui Hui’s group, Guangzhou University of Chinese Medicine, Xuwen, Guangdong (Figure 1) [8]. According to the literature comparison of spectral and physical data of the compound, compound **7** was identified as diaporthein B (DTB) [8]. Currently, there are reports on the antibacterial [8] and anti-tumor [9] properties of DTB; however, no reports on its effects in the inhibition of colon cancer exist. In this research, DTB was found to have a significant inhibitory effect on colon cancer, and its mechanism was preliminarily discussed.

## 2. Results

### 2.1. Inhibition of Colon Cancer Cell Proliferation by DTB

**Figure 2:**
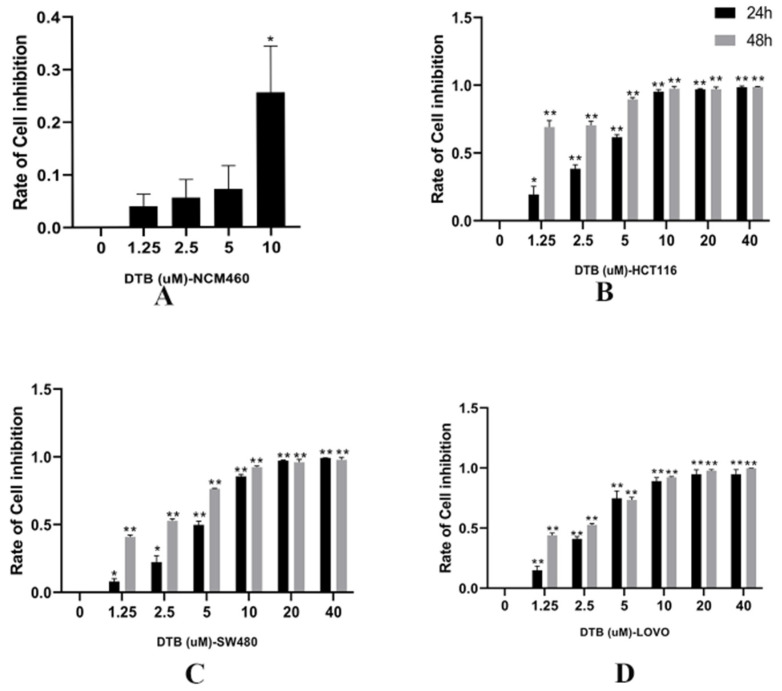
(**A**) The 24 h dose–response curves of DTB on normal human intestinal epithelial cells NCM460. (**B**) The dose–response curves of DTB on HCT116 colon cancer cells for 24 and 48 h. (**C**) The dose-response curves of DTB on SW480 colon cancer cells for 24 and 48 h. (**D**) The dose-response curves of DTB on LOVO colon cancer cells for 24 and 48 h. The abscissa is the DTB concentration and the ordinate is the rate of cell inhibition with the blank control group as a reference. *n* = 4; each data point represents the mean ± standard error. Compared with the blank group, * *p* < 0.05, ** *p* < 0.01.

Compared with the blank group, 1.25, 2.5, and 5 μmol/L of DTB had no significant effect on human normal intestinal epithelial cell NCM460 (*p* > 0.05, with no statistical significance (Figure 2A). Further, compared with the blank group, the inhibition rates of HCT116, SW480, and LoVo of three human colon cancer cells were significantly changed after 24 and 48 h of DTB intervention with different concentrations (Figure 2B–D). DTB inhibited the proliferation of three kinds of colon cancer cells in a dose-dependent and time-dependent manner (*p* < 0.05), which was statistically significant. The IC50 (half maximal inhibitory concentration) concentrations of DTB on HCT116, SW480, and LOVO colon cancer cells for 24 h were 3.14, 4.55, and 3.08 μmol/L, respectively, whereas the IC50 concentrations for 48 h were 0.75, 1.90, and 1.89 μmol/L, respectively. According to the above results, HCT116 and LOVO cells were selected for follow-up experiments. The medication time was 24 h, and the concentrations were 1.5 and 3 μmol/L, respectively.

### 2.2. DTB Inhibits Cell Clonogenic Ability

**Figure 3:**
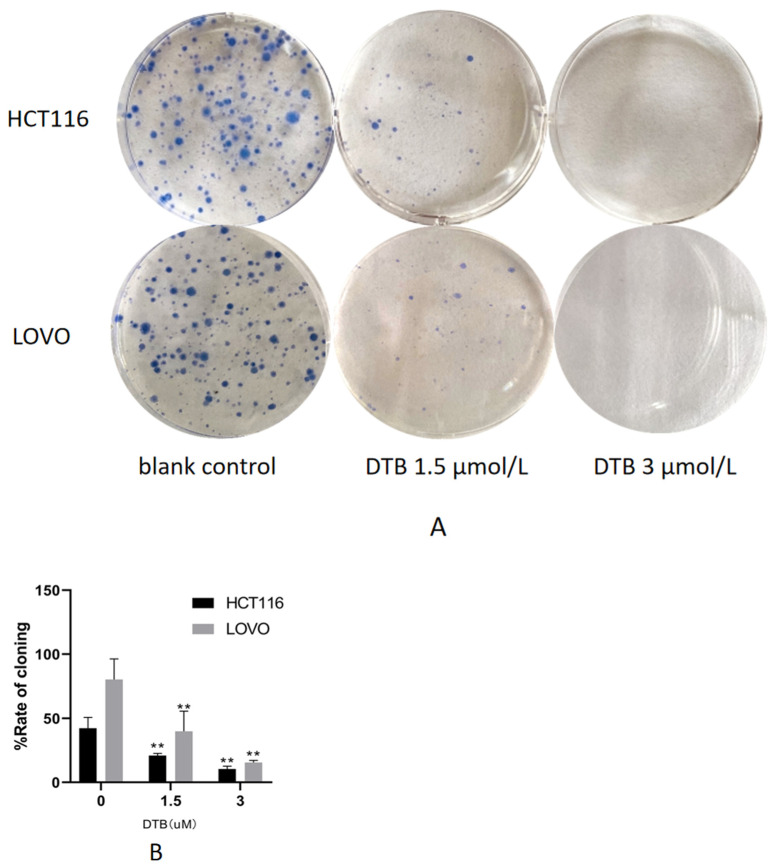
(**A**) The effect of DTB on colon cancer HCT116 and LOVO cells’ clone formation ability. The left is the blank control group, the middle is the DTB 1.5 μmol/L group, and the right is the DTB 3 μmol/L group. (**B**) Rate of clone formation. *n* = 3; each data point represents the mean ± standard error. Compared with the blank group, ** *p* < 0.01.

The colony formation test is one of the effective methods to measure the ability of cells to proliferate. The adherent cells may not be able to proliferate and form clones, but the cells that form clones must be adherent and proliferative cells. Compared with the blank group, the clonogenic rate of HCT116 and LOVO cells was significantly decreased, and the clonogenic effect was enhanced with the increase in DTB concentration (Figure 3). As the concentration of DTB drug increased, its effect of inhibiting clonogenicity also increased.

### 2.3. DTB Can Inhibit Migration of Colon Cancer Cells

**Figure 4:**
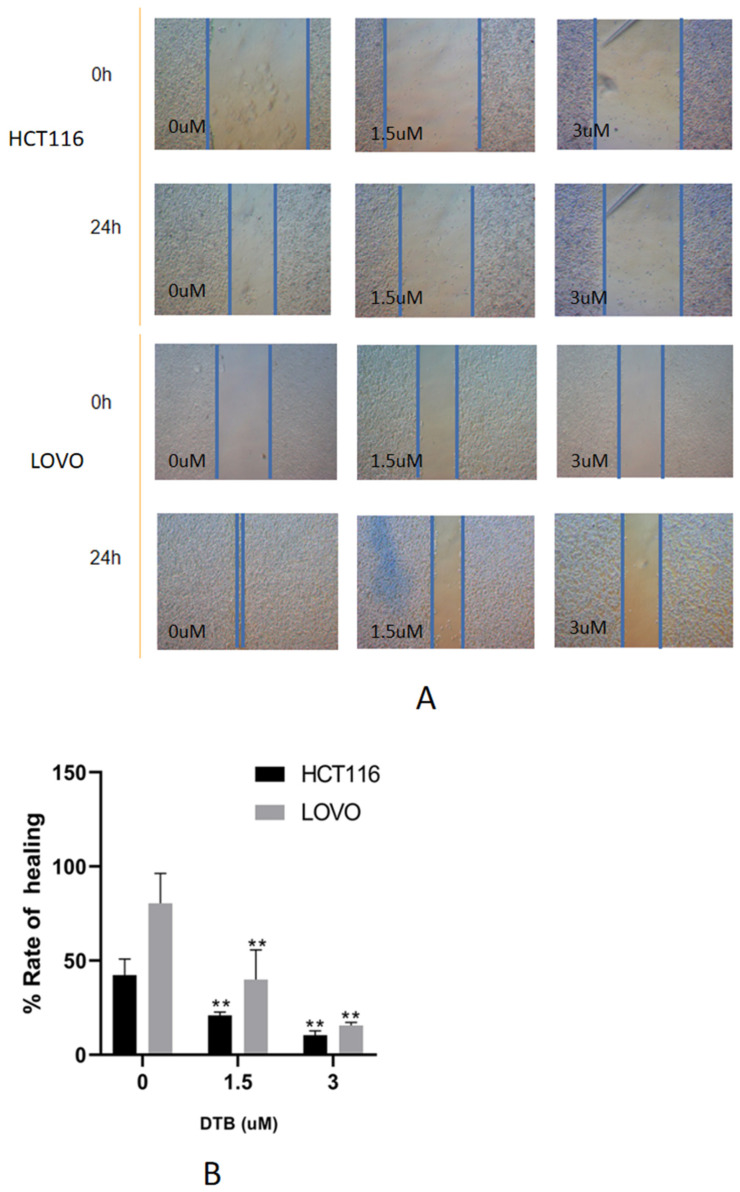
(**A**) The effect of different concentrations of DTB on the wound healing of HCT116 and LOVO cells under the microscope (×40). (**B**) The effect of DTB on the wound healing rate of colon cancer. *n* = 3; each data point represents the mean ± standard error. Compared with the blank group, ** *p* < 0.01.

Compared with the blank group, the wound healing rate of HCT116 and LOVO cells was significantly decreased. The healing rate decreased with the increase in DTB concentration, suggesting that DTB can inhibit the migration of colon cancer cells (Figure 4). The dynamic reorganization of the cytoskeleton, the adhesion changes between the extracellular matrices, and the dynamic changes in the surrounding matrix can all affect the migration ability of cells. These responses are regulated by complex signals, among which focal adhesion [10], integrin [11], focal adhesion kinase [12], Rho family guanosine triphosphatase (GTPases) proteins [13] play important roles. Therefore, the decrease in cell migration ability may be related to the above signals, which may involve MAPKs (mitogen activated protein kinases), PI3K (phosphatidylinositol 3-kinase), and other signaling pathways.

### 2.4. Apoptotic Morphology Was Assessed by Fluorescence Microscopy

**Figure 5:**
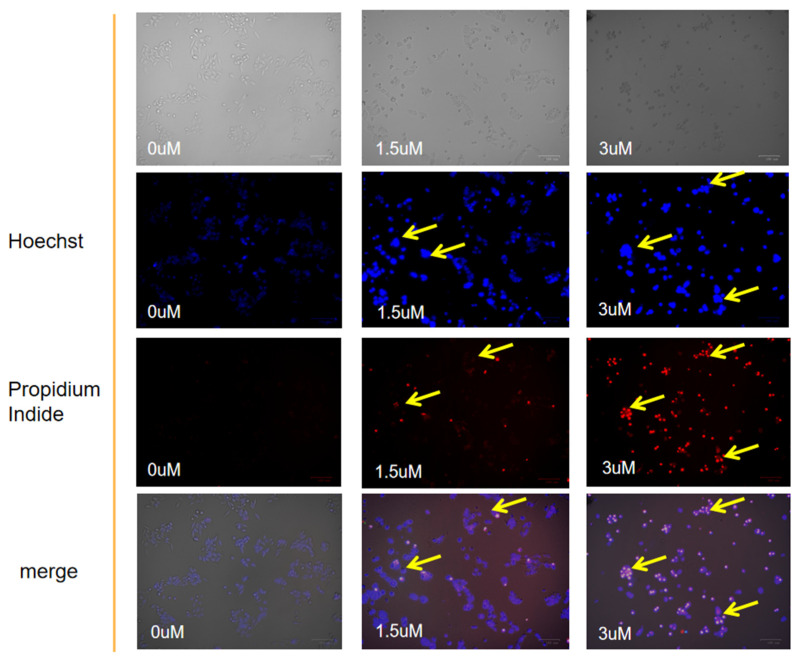
Hoechst 33342 and PI staining to observe the effect of DTB on the apoptotic morphology of human colon cancer LOVO cells. The bright blue and red coloring can be seen at the place indicated by the yellow arrow.

Compared with the blank group, the proportion of apoptosis in the DTB group (5 and 3 μmol/L) was significantly increased, with red staining showing late apoptotic or necrotic cells, and bright blue staining showing mid-early apoptotic cells with fragmented, lobulated, and edge-set nuclei. The bright blue and red stains are evident at the arrow (Figure 5), indicating that colon cancer cells are in a state of apoptosis or necrosis. In the merge diagram, normal cells are low blue and low red, apoptotic cells are high blue and low red, and necrotic cells are low blue and high red. Furthermore, the proportion of apoptosis in the DTB group was higher at concentrations 3 μmol/L and 1.5 μmol/L. With the morphological changes of apoptosis shown by Hoechst 33342 and PI (Propidium Iodide) staining, it was observed that DTB could induce the apoptosis of colon cancer cells.

### 2.5. Apoptosis Was Assessed by Flow Cytometry

**Figure 6:**
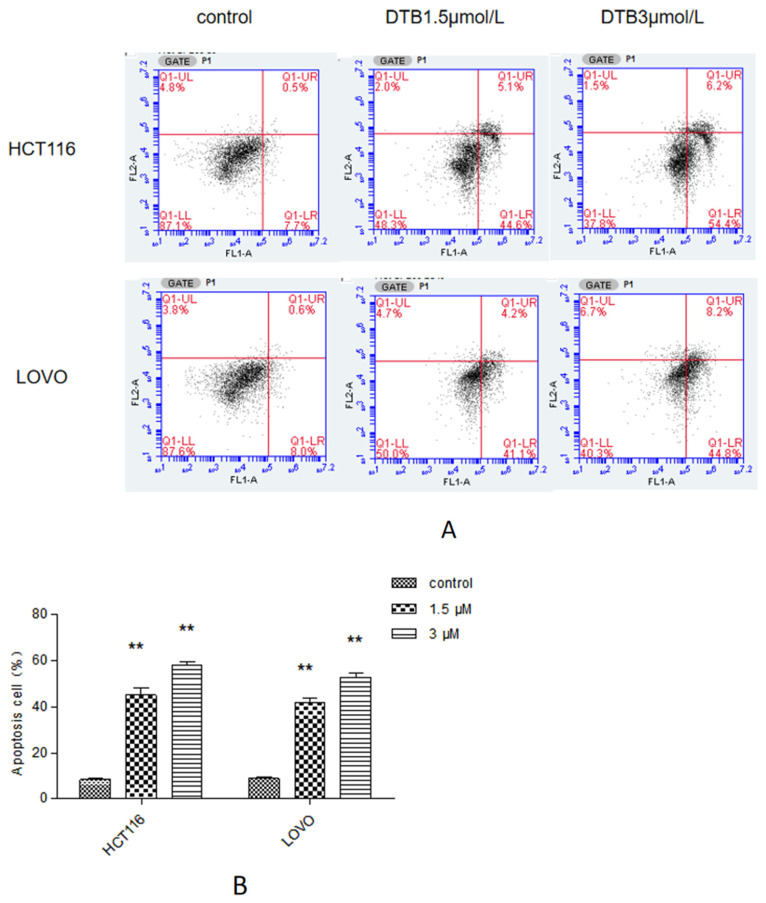
(**A**) Flow cytometry to detect the apoptosis rate of colon cancer cells HCT116 and LOVO after DTB intervention. (Q1-UR: the late apoptotic cells; Q1-LR: the early apoptotic cells. The abscissa is Annexin V FITC staining, and the ordinate is PI staining.) (**B**) Flow cytometric detection of the apoptotic rate of colon cancer cells. *n* = 3; compared with the blank group, ** *p* < 0.01, each data point represents the mean ± standard deviation.

Compared with the blank group, the percentage of apoptotic cells in 1.5 μmol/L and 3 μmol/L DTB groups increased significantly, and the proportion of early apoptotic cells increased more as compared with those in the late apoptotic cells. The percentage of apoptotic cells in the 3 μmol/L DBT group also showed a higher trend than those in the 1.5 μmol/L DBT group (Figure 6).

### 2.6. Detection of Target Gene by RT-PCR

**Figure 7:**
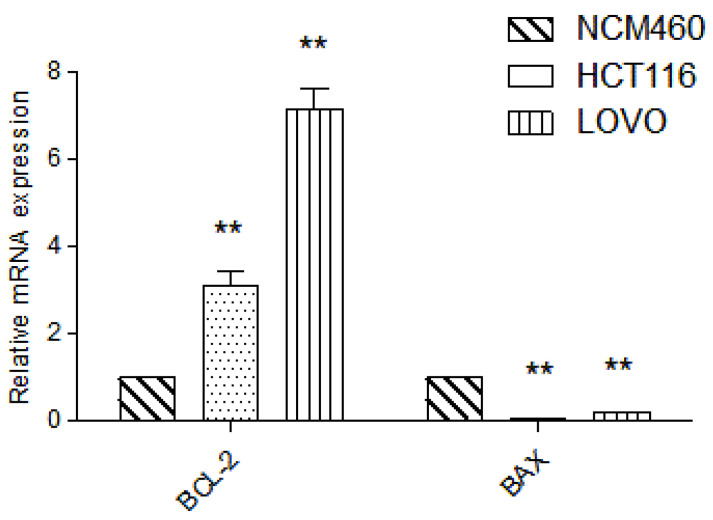
Changes in the expression of mitochondrial apoptosis-related genes BCL-2 and BAX in human normal intestinal epithelial cells NCM460 and colon cancer cells HCT116 and LOVO. *n* = 3; Compared with the blank group, ** *p* < 0.01, each data point represents the mean ± standard error.

**Figure 8:**
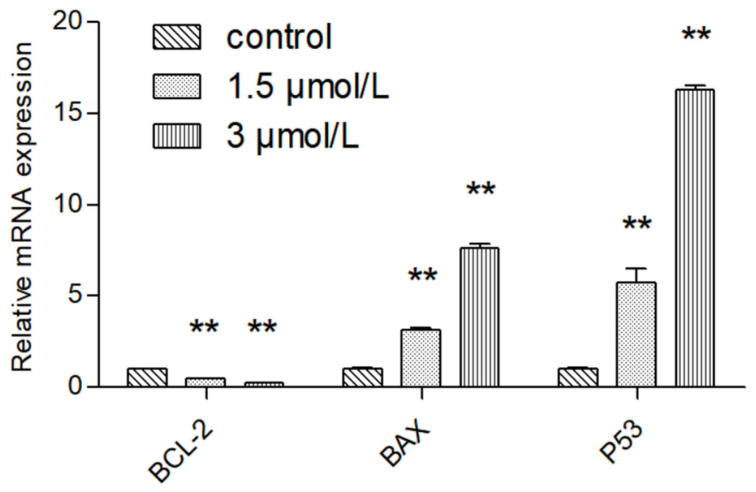
The effect of DTB on the expression of mitochondrial-related genes BCL-2, BAX, and TP53 in colon cancer cells LOVO. *n* = 3; compared with the blank group, ** *p* < 0.01, each data point represents the mean ± standard error.

**Figure 9:**
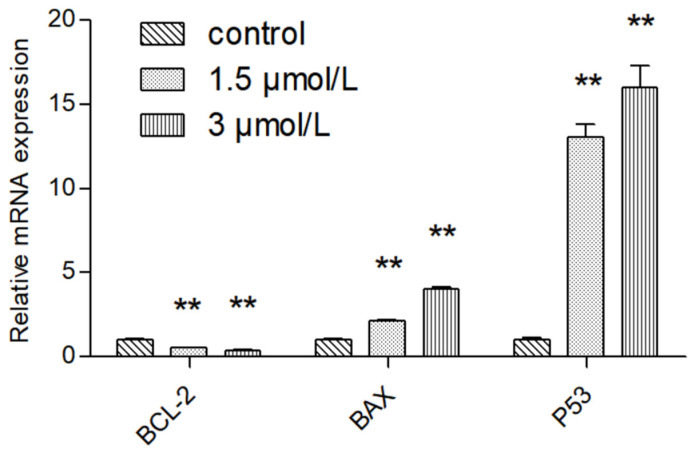
The effect of DTB on the expression of mitochondrial-related genes BCL-2, BAX, and TP53 in colon cancer cells HCT 116. *n* = 3; compared with the blank group, ** *p* < 0.01, each data point represents the mean ± standard error.

**Figure 10:**
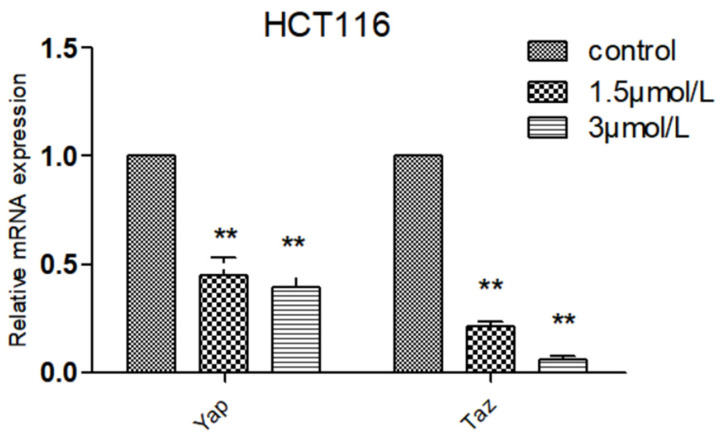
The effect of DTB on the expression of YAP and TAZ related genes of the HCT116 Hippo pathway in colon cancer cells. *n* = 3; compared with the blank group, ** *p* < 0.01, each data point represents the mean ± standard error.

**Figure 11:**
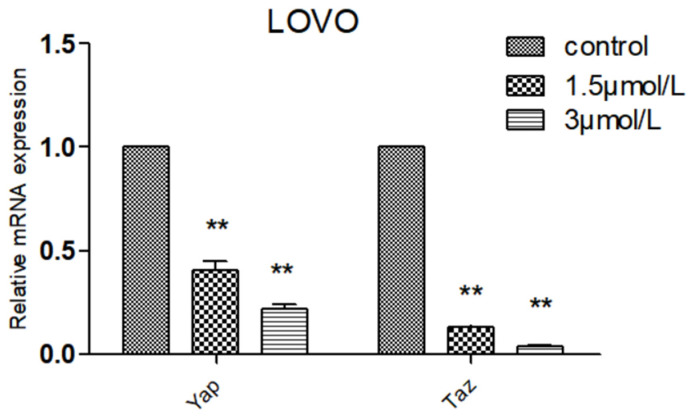
The effect of DTB on the expression of YAP and TAZ related genes of the LOVO Hippo pathway in colon cancer cells. *n* = 3; compared with the blank group, ** *p* < 0.01, each data point represents the mean ± standard error.

Compared with normal intestinal epithelial cells, the mRNA (Messenger RNA) expression of apoptosis-related gene BCL-2 (B-cell lymphoma-2) was significantly increased (*p* < 0.01) and the mRNA expression of Pro-apoptotic gene BAX (BCL2-Associated X) was significantly decreased (*p* < 0.01) in colon cancer HCT116 and LOVO cells, indicating the high expression of Bcl-2 and low expression of BAX in colon cancer cells, which was consistent with the literature (Figure 7). Compared with the blank group, the expression of mitochondrial apoptosis-related gene BCL-2 mRNA in the DTB group of HCT116 and LOVO cells was significantly decreased (*p* < 0.01), and the expression of pro-apoptotic gene BAX and tumor suppressor gene TP53 (tumor protein p53) mRNA was significantly upregulated (*p* < 0.01). These results showed that DTB could regulate the mitochondrial apoptotic TP53/BCL-2/BAX signaling pathway in colon cancer cells (Figure 8 and Figure 9). Compared with the blank group, the mRNA expression of Hippo signaling pathway-related genes YAP (Yes associated protein) and TAZ (Tafazzin) in the DTB group of colon cancer cells HCT116 and LOVO was significantly decreased (*p* < 0.01). These results suggested that DTB may promote apoptosis by crosstalk with the mitochondrial apoptotic TP53/BCL-2/BAX signaling pathway via two key factors of the Hippo signaling pathway YAP and TAZ (Figure 10 and Figure 11).

### 2.7. Effect of DTB on Hippo Signaling Pathway Effectors YAP and TAZ

**Figure 12:**
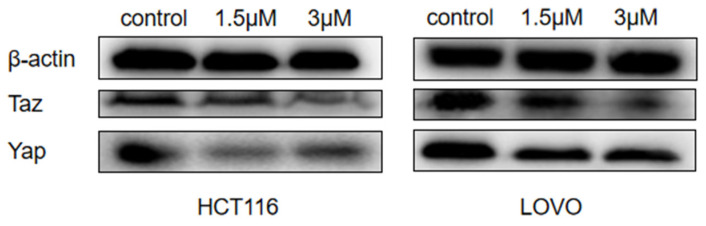
Electrophoresis of Yaz and TAZ protein expression in colon cancer cells after DTB intervention.

**Figure 13:**
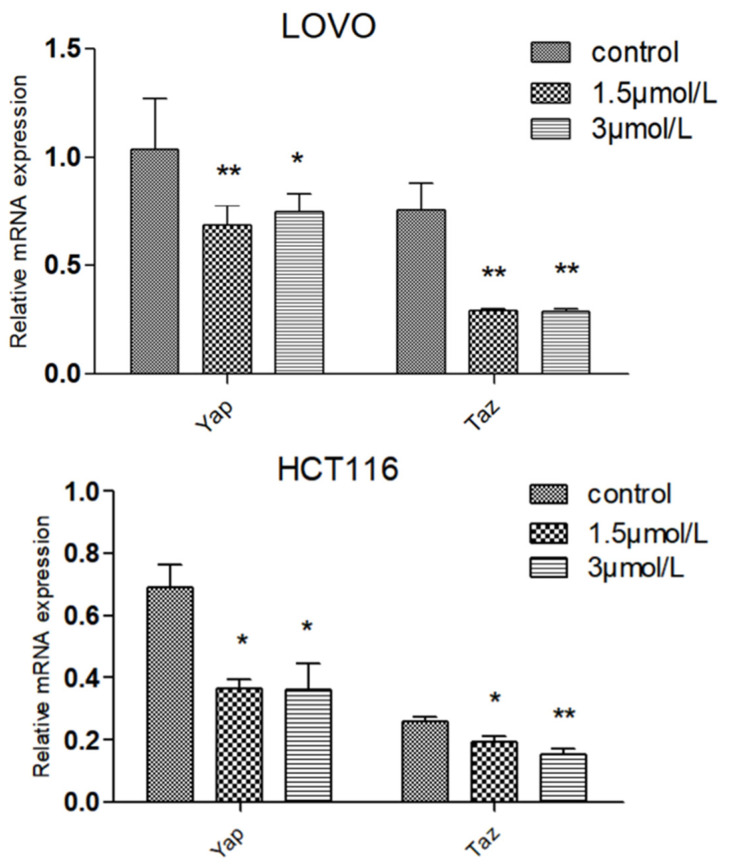
Electrophoretic grayscale values of Yaz and TAZ protein expression in colon cancer cells after DTB intervention. *n* = 3; compared with the blank group, * *p* < 0.05; ** *p* < 0.01, each data point represents the mean ± standard error.

The expression of YAP and TAZ proteins was significantly decreased in colon cancer cell HCT116 (*p* < 0.01), and the expression of YAP and TAZ proteins was also significantly decreased in colon cancer cell LOVO (*p* < 0.01), indicating that DTB can indeed inhibit the elevation of YAP and TAZ, possibly by decreasing the association of YAP and TAZ with transcription factors in the nucleus or crosstalk with the cellular mitochondrial apoptosis pathway, thereby inducing apoptosis of colon cancer cells (Figure 12 and Figure 13).

## 3. Discussion

Although significant progress has been made in the treatment of colon cancer, a high mortality rate still persists, which demonstrates the limitations of conventional therapies. For patients treated with postoperative chemotherapy and who experience postoperative recurrence, drugs that carry large toxic side effects tend to develop drug resistance. Therefore, the exploration of effective means to treat colon cancer, enhance the efficacy of drugs, and reduce the toxic side effects of drugs are the current research hotspots.

DTB has good antibacterial and anti-tumor activities. Its effects have been validated in a variety of tumor cells. Based on these results, it can be speculated that DTB may have the potential to develop into a broad-spectrum anti-tumor chemotherapeutic agent. However, the current research on DTB is limited, and there are no relevant research reports describing its effects on colon cancer inhibition. Moreover, most studies only evaluated its broad-spectrum anti-tumor effect on the inhibition and proliferation of cancer cells without further exploring its mechanism.

In the present study, we found that DTB could inhibit the proliferation of three colon cancer cells, HCT116, SW480, and LOVO, but had no obvious effect on normal human intestinal epithelial cells. In further research, DTB was found to promote the apoptosis of HCT116 and LOVO colon cancer cells, with similar sensitivity to DTB.

TP53, as a tumor suppressor gene, transcriptionally controls hundreds of genes, which are involved in regulating cell proliferation, DNA damage repair, and apoptosis induction [14,15,16,17], and is an important apoptosis-regulating protein [18]. TP53 inhibits or activates anti-apoptotic factors (BCL-2, BCL-2-XL, BCL-2-XW, and MCL-1) [19] or pro-apoptotic effectors (BAX, BAK, BID, BIM, BIK, BMF, BIF-1, BAD, NOXA, and HRK) [20,21,22] through the DBD domain and Hippo pathway, respectively, and promotes the apoptosis of cancer cells. TP53 (P53) is an important tumor suppressor gene, and mutations in the P53 gene have been reported to be found in up to 89% of colon cancer patients [23]. Activation of TP53 can downregulate the expression of BCL-2, activate BAX, and induce apoptosis. The key regulators in the process of apoptosis are BCL-2 family proteins, which mainly include anti-apoptotic regulators (such as BCL-2) and pro-apoptotic regulators (such as BAX) [24]. In the mitochondrial pathway, cytochrome C is released into the cytoplasm through the mitochondrial PT pore (permeability transition pore) or the mitochondrial transmembrane channel formed by members of the BCL-2 family. BCL-2 family proteins play a key role in regulating the opening and closing of PT pores. Pro-apoptotic proteins such as BAX can mediate the opening of PT pores by binding to ANT, while anti-apoptotic proteins such as BCL-2 can compete with BAX. It can bind to ANT or directly organize the binding of BAX and ANT to exert anti-apoptotic effects [25]. BCL-2 can form a dimer with BAX. If the relative amount of BAX is higher than that of BCL-2, the number of BAX homodimers will increase, thereby promoting cell death [26]. If the relative amount of BCL-2 is higher than that of BAX, it promotes the formation of BCL-2/BAX heterodimer and increases the amount of BCL-2 homodimer, thereby inhibiting cell death. Studies have shown that curcumin can induce apoptosis in LOVO cells by regulating the expression of BCL-2 family proteins [27]; sorbitol can induce apoptosis by activating the mitochondrial pathway of apoptosis by upregulating BAX and downregulating BCL-2 [28].

The Hippo pathway is a key regulator of the physiological process of development and regeneration, but if the regulation is relaxed, it will induce cell transformation and cancer progression. The YAP/TAZ gene is a key effector of the Hippo signaling pathway. YAP/TAZ is amplified and preferentially localized in the nuclei of several tumors: the lung, pancreas, esophagus, stomach, skin, colon, prostate cancer, liver cancer, ovarian cancer and breast cancer medulloblastoma, glioma, and oral squamous cell carcinoma [29,30,31]. The Hippo signaling pathway interacts with many other signaling pathways to form a complex network, in which YAP/TAZ is a key node to integrate and decode tumor inhibition [29,30,32].

Research has shown that TP53 and Hippo signaling pathways are functionally connected, and the Hippo pathway and TP53, as tumor suppressors, synergistically induce aging and apoptosis. YAP can induce the expression of P21, BAX, and Caspase 3 by combining with the TP53 promoter and inhibiting the expression of anti-apoptotic factors BCL-2 and BCL-2-XL, resulting in cell cycle arrest and apoptosis induction [33]. YAP induces TP53 transcription, which in turn binds to the promoter of YAP and activates YAP transcription in a positive feedback loop [34]. Furthermore, at the protein level, researchers demonstrated that YAP was overexpressed in 28 colorectal cancer samples [34], and by mining the Cancer Proteome Atlas [35], we found that YAP and TAZ were overexpressed in colon cancer. In human colon cancer cell experiments, after YAP was silenced by shRNA, cell proliferation, metastasis, and invasion were significantly reduced, while YAP overexpression led to colon cancer cell proliferation [36,37]. Since the crossover between TP53 and the Hippo signaling pathway can induce both tumor suppressive and carcinogenic effects, its targeted therapy may have great potential in the treatment of human cancer.

Based on this, this study further explored the mechanism of DTB promoting the apoptosis of colon cancer cells and found that DTB can promote the expression of TP53 in colon cancer cells HCT116 and LOVO, indicating that DTB may activate the TP53 pathway of colon cancer cells. Therefore, in this research, the mRNA profiles of BCL-2, an anti-apoptotic regulator, and BAX, a pro-apoptotic regulator, two important factors in the mitochondrial apoptotic signaling pathway were examined. It was found that the expression of BCL-2 mRNA was significantly decreased, while the expression of BAX mRNA was significantly increased in colon cancer cells HCT116 and LOVO, indicating that DTB was most likely to promote colon cancer cell apoptosis by activating the mitochondrial apoptotic signaling pathway. In summary, it was considered that DTB may activate the mitochondrial apoptosis pathway of BAX/BCL-2/TP53. In the subsequent experiment, we examined the mRNA levels and protein levels of YAP and TAZ in colon cancer cells HCT116 and LOVO after DTB intervention. It was found that after DBT intervention, the mRNA expression and protein expression of YAP and TAZ, two key factors of the Hippo signaling pathway, were significantly decreased, indicating that DTB was most likely to reduce the binding with transcription factors in the nucleus by downregulating YAP and TAZ expression, thus promoting cells apoptosis through the Hippo signaling pathway. DTB has good anti-colon cancer activity, has no obvious effect on normal intestinal epithelial cells, and may have the potential to be developed as an anti-colon cancer chemotherapy drug.

## 4. Materials and Methods

### 4.1. Experimental Materials

#### 4.1.1. Cells

HCT116 cells, LOVO cells, SW480 cells, and NCM460 cells were purchased from ATCC (American type culture collection, Manassas, VA, USA) and deposited in the Central Laboratory of basic research of integrated traditional Chinese and Western medicine, School of Basic Medical Sciences, Guangzhou University of Chinese Medicine.

#### 4.1.2. Medicine

Prof. Cui Hui, School of Pharmaceutical Sciences, Guangzhou University of Chinese Medicine, and his research group isolated the marine fungus Penicillium sclerotiorum GZU-XW03-2 from the intestinal tract of the Onchidium struma, collected from Xuwen, Guangdong. The compound DTB [8] was obtained from the EtOAc extract of its fermentation broth through molecular biological methods. According to molecular biological methods, DNA amplification and ITS region sequencing were performed (registered in GenBank, accession No.: MT071304). The strain is currently stored in the School of Pharmaceutical Sciences, Guangzhou University of Chinese Medicine, China, with the access code, 2018-GZU-XW0-2.

#### 4.1.3. Experiment Reagents

Fetal bovine serum, DMEM high-glucose medium, RPMI1640 medium, cyan streptomycin, 0.25% trypsin, phosphate buffered saline (PBS), and 0.25% EDTA free trypsin were purchased from GIBCO, Grand Island, CA, USA. Dimethyl sulfoxide (DMSO) was purchased from Sigma, Rockville, MD, USA. The CCK8 kit was purchased from Dalian Meilun Biotechnology Co., Ltd., Dalian, China. FITC annexin V and PI apoptosis kits were purchased from Genecopoeia, Rockville, MD, USA. Four percent paraformaldehyde was purchased from Guangzhou Ruishu biotechnology Co., Guangzhou, China. Giemsa staining solution was purchased from Beijing Dingguo Changsheng Biotechnology, Bejing, China. Rainbow 180 broad spectrum protein marker (11–180 KD) was purchased from Beijing Solai Bao Technology Co., Ltd., Beijing, China. Hoechst 33342 stain, propidium iodide (PI), 4% SDS-PAGE supergel master mix, 10% SDS-PAGE supergel master mix, Western primary antibody diluent, Western secondary antibody diluent, and beyoecl moon (extreme ultra ECL chemiluminescence kit, 5 × Loading buffer, and Tween-20), were purchased from Beyotime Biotechnology (Shanghai, China). Pierce BCA protein assay kit was from Thermo, Waltham, MA, USA. RNA Purification Kit was purchased from Ezbioscience, Roseville, MN, USA. Primescript RT master mix and TB green premixex TAQ (TLI RNASEH plus) were purchased from Takara, Kusatsu City, Japan. Absolute ethanol and 95% ethanol were purchased from Guangzhou Chemical Reagent Factory, Guangzhou, China. Methanol and glycine were purchased from Shanghai Macklin Biochemical Technology Co., Ltd., Shanghai, China. Sodium dodecyl sulfate (SDS), protease inhibitor cocktail, Ripa lysis buffer, and PMSF were purchased from Hangzhou Verde Bio Technology Co., Ltd., Hangzhou, China. Tris was purchased from Guangzhou Saiguo Bio Technology Co., Ltd., Guangzhou, China. Ammonium persulfate (APS) was purchased from Tianjun Biotech Co., Ltd., Tianjin, China. TEMED was purchased from Shanghai SANGON Co., Ltd., Shanghai, China. Nonfat dry milk was purchased from Becton Dickinson, Franklin Lakes, NJ, USA. Beta actin antibody, YAP antibody, TAZ antibody, and Goat anti-rabbit IgG were purchased from Affinity, West Bridgford, UA. Cultured cells were then incubated in an incubator (at 37 °C and 5.0% CO_2_ atmosphere).

### 4.2. Experiment Method

#### 4.2.1. Drug Dissolution

An appropriate amount of DTB powder was weighed through an electronic balance, the corresponding volume of DMSO solution was added, fully dissolved into mother liquor with a concentration of 50 mmol/L, and placed in the refrigerator at −20 °C for storage. When the reagent was needed in the experiment, it was used in the required concentrations. This reagent is ready for use as soon as it is prepared.

#### 4.2.2. CCK-8 Experiment

HCT116, SW480, LOVO, and NCM460 cells in the logarithmic growth stage were inoculated into a 96-well culture plate at the density of 5.0 × 10^3^ cells per well. Colon cancer cells (0, 1.25, 2.5, 5, 10, 20, and 40 μmol/L) were divided into nine groups with four multiple pores in each group. NCM460 cells were divided into seven groups: blank group (no cells), blank control group (no drugs), and DTB experimental group (0, 1.25, 2.5, 5, and 10 μmol/L). The 96-well plate was incubated overnight. The blank control group was replaced with a culture medium, and the DTB experimental group was replaced with a culture medium supplemented with preset concentrations and incubated for 24 and 48 h, respectively. Ten microliters of CCK-8 solution were added per well and incubated for 2 h; thereafter, the absorbance OD value of 450 nm was measured by a multifunctional microplate reader. The cell survival rate of the blank group was taken as 100% to calculate the cell survival rate of other groups and draw the survival rate of the cells. IBM SPSS statistical software was used to calculate the 95% confidence interval of IC50, and cells, drug time, and concentration were screened for subsequent experiments.

#### 4.2.3. Plate Cloning Experiment

According to the CCK-8 results, HCT116 and LOVO cells at the logarithmic growth stage were inoculated into the 6-well cell culture plate at a density of 300 cells per well and incubated overnight. Three groups were made: blank control group and DTB experimental group (1.5 and 3 μmol/L). The blank group was replaced with culture medium, and the experimental group was replaced with the preset DTB concentrations of 1.5 μmol/L and 3 μmol/L, respectively. The corresponding concentration of the medium was changed every 3 days to maintain the concentration of drug administration, continuous culture for 2–3 weeks. Culture was terminated when naked eye clones appear in a Petri dish. The culture medium was discarded, and 4% paraformaldehyde was added to fix the cells for 15 min; then, the fixing medium was removed, and washed with PBS twice; 800 μL Giemsa dye solution was added to the well for 20–30 min, and the dye solution was washed off slowly with running water and dried at room temperature. More than 10 cells were photographed under a microscope (low power microscope), and the number of cell clusters was counted. The cloning efficiency of cells in other groups was calculated by taking the cloning rate of the blank group as 100%.

#### 4.2.4. Wound Healing Assay

Under ultraviolet irradiation, the ruler and marker were placed in the ultra-clean table for 30 min, and five horizontal lines were evenly drawn with a marker behind the 6-hole plate compared with the ruler, and each line was separated by 0.5–1 cm to cross the hole. The cells were inoculated on 6-well plates with a density of 8 × 10^5^·well^−1^, and there were three groups, including blank control group and DTB experimental group (1.5 and 3 μmol/L), with three wells in each group, and incubated overnight. After the battery was fully charged, the gun head was compared with a ruler and scratch perpendicular to the horizontal line behind the 6-hole plate. After scratching, PBS was washed three times. The blank group was replaced with a serum-free medium. The experimental group was given DTB, and the final concentration was 1.5 μmol/L and 3 μmol/L, respectively. Serum-free medium was added and incubated in the incubator. According to 0, 6, 12, and 24 h photography, the wound healing rate was calculated by Image J (Rawak Software, Inc., Stuttgart, Germany).

#### 4.2.5. Hoechst 33342/PI Staining Experiments

HCT116 and LOVO cells at the logarithmic growth stage were inoculated in 6-well plates at a density of 5 × 10^5^ well^−1^ and incubated overnight. The blank control and DTB experimental groups (1.5, 3 μmol/L) were divided into 3 groups with 3 rewells in each group. The blank group was replaced with a complete culture medium, and the experimental group was given DTB with final concentrations of 1.5 μmol/L and 3 μmol/L, respectively. The cells were incubated in a 3 μmol/L culture medium for 24. The supernatant was discarded and washed twice with PBS. Then, 2 mL of culture solution, 2 μL Hoechst 33342 staining solution, and 2 μL PI staining solution were added to each well, dyed on shaking table for 10 min. Then, the staining solution was discarded, sterile PBS was added and washed twice immediately to observe under the fluorescence microscope and take photos for recording.

#### 4.2.6. Flow Cytometry

HCT116 and LOVO cells at the logarithmic growth stage were inoculated in 6-well plates at a density of 2 × 10^5^ well^−1^ and incubated overnight. The blank control and DTB experimental groups (1.5, 3 μmol/L) were divided into 3 groups with 3 rewells in each group. The blank group was replaced with a complete culture medium, and the experimental group was given DTB with final concentrations of 1.5 μmol/L and 3 μmol/L, respectively, for 24 h intervention. Cells were collected and stained according to the instructions for the FITC Annexin V and PI Apoptosis Kit. The experimental data were exported and processed by BD CSampler software (Becton, Dickinson and Company, Franklin lakes, NJ, USA).

#### 4.2.7. Real-Time PCR

HCT116 and LOVO cells at the logarithmic growth stage were inoculated in 6-well plates at a density of 2 × 10^5^ well^−1^ and incubated overnight. The blank control and DTB experimental groups (1.5, 3 μmol/L) were divided into 3 groups with 3 rewells in each group. The blank group was replaced with culture medium, and the experimental group was given DTB culture medium with the final concentrations of 1.5 μmol/L and 3 μmol/L, respectively, and incubated for 24 h. The supernatant was discarded and washed twice with PBS. The culture medium was discarded, and RNA was extracted according to the instructions of the EZB-Press RNA Purification Kit. The extracted RNA was placed on ice and reverse-transcribed into cDNA according to the instructions of the reverse transcription kit. Then, according to the instructions of TB green Premix Ex Taq kit, it was amplified by PCR instrument that was used to amplify the data, and 2^−ΔΔCt^ method was used to analyze the exported data.

#### 4.2.8. Western Blot

Total protein extract was prepared by Western Blot. Logarithmic growth HCT116 and LOVO cells were inoculated into 6-well plates at a density of 2 × 10^5^ well^−1^ and inoculated overnight. The blank control and DTB experimental groups (1.5, 3 μmol/L) were divided into 3 groups with 3 rewells in each group. The blank group was replaced with a complete culture medium, and the experimental group was given DTB with final concentrations of 1.5 μmol/L and 3 μmol/L, respectively, for 24 h intervention. The cells were collected, the protein concentration was measured by the BCA method, the samples were loaded, the proteins were separated by polyacrylamide gel electrophoresis and then transferred to the membrane. The sample was sealed with skimmed milk sealing solution for 2 h, YAP (1:1000), TAZ (1:1000), β-Actin (1:1500) primary antibody was added and incubated at 4 °C overnight. The next day, after washing the membrane, goat anti-rabbit IgG (1:2000) secondary antibody was added and incubated in a shaking table at room temperature for 2 h. After washing the film, it was exposed according to ECL luminous solution instructions. After the exposed pictures were exported, Image J was used to measure the gray level. Based on the internal parameters, the relative gray values were calculated using other values and their ratios.

#### 4.2.9. Statistical Approach

IBM SPSS statistics 21.0 (IBM, Armonk, NY, USA) statistical software was used for analysis, and all data were tested for normal distribution. The Kruskal–Wallis method was used for statistical analysis of data that did not conform to normal distribution. Single factor analysis was used for normal distribution and homogeneity of variance, and LSD was used for pairwise comparison between groups. The robustness test for variance with equal means was selected, and the Dunnett-t method was used for pairwise comparison between groups. *p* < 0.05 was considered statistically significant.

## 5. Conclusions

Based on the above results, we concluded that DTB significantly inhibited the proliferation and migration of colon cancer. Since Hippo signaling mostly experiences crosstalk with other signaling pathways, it can be inferred that crosstalk may be formed by activating the TP53/BCL-2/BAX pathway and Hippo/YAP/TAZ signaling pathway. The whole process may lead to impaired mitochondrial function through key factors of the Hippo signaling pathway YAP and TAZ and the tumor suppressor gene TP53, thus ultimately promoting apoptosis of colon cancer cells. This research paved the way for the application and mechanism of DTB in the treatment of colon cancer and laid a research foundation for the development and application of DTB as an anti-colon cancer drug.

## Data Availability

Data are contained within the article.

## Sample Availability

Samples of the compound DTB are available from the authors.
